# Plasma Branched-Chain Amino Acids Are Associated With Greater Fasting and Postprandial Insulin Secretion in Non-diabetic Chinese Adults

**DOI:** 10.3389/fnut.2021.664939

**Published:** 2021-04-28

**Authors:** Cherlyn Ding, Leonie Egli, Nabil Bosco, Lijuan Sun, Hui Jen Goh, Khung Keong Yeo, Jonathan Jiunn Liang Yap, Lucas Actis-Goretta, Melvin Khee-Shing Leow, Faidon Magkos

**Affiliations:** ^1^Nestlé Research, Singapore, Singapore; ^2^Nestlé Institute of Health Sciences, Nestlé Research, Lausanne, Switzerland; ^3^Singapore Institute for Clinical Sciences, Singapore, Singapore; ^4^Duke-NUS Medical School, Singapore, Singapore; ^5^National Heart Centre Singapore, Singapore, Singapore; ^6^Lee Kong Chian School of Medicine, Nanyang Technological University, Singapore, Singapore; ^7^Yong Loo Lin School of Medicine, National University of Singapore, Singapore, Singapore; ^8^Department of Endocrinology, Tan Tock Seng Hospital, Singapore, Singapore; ^9^Department of Nutrition, Exercise & Sports, University of Copenhagen, Frederiksberg, Denmark

**Keywords:** BCAA, insulin resistance, insulin metabolism, glucose homeostasis, insulin clearance

## Abstract

**Background:** Plasma branched-chain amino acids (BCAA) are consistently elevated in subjects with obesity and type 2 diabetes (T2DM) and correlate with insulin resistance. The association of BCAA with insulin secretion and clearance rates has not been adequately described.

**Objective:** To evaluate the relationships between fasting and postprandial plasma BCAA, insulin secretion and insulin clearance.

**Design:** Ninety-five non-diabetic Chinese subjects (43 females) underwent a mixed-meal tolerance test; blood biomarkers including BCAAs (leucine, isoleucine, valine) were measured for 6 h. Fasting and postprandial insulin secretion rates (ISR) and insulin clearance were determined by oral minimal modeling of glucose and C-peptide.

**Results:** Fasting and postprandial plasma BCAA correlated strongly with each other (ρ = 0.796, *P* < 0.001), and both were positively associated with basal ISR (ρ = 0.45/0.36, *P* < 0.001), total postprandial ISR AUC (ρ = 0.37/0.45, *P* < 0.001), and negatively with insulin clearance (ρ = −0.29/−0.29, *P* < 0.01), after adjusting for sex and body mass index. These relationships largely persisted after adjusting further for insulin resistance and postprandial glucose. Compared with subjects in the middle and lowest tertiles for fasting or postprandial plasma BCAA, subjects in the highest tertile had significantly greater postprandial glucose (by 7–10%) and insulin (by 74–98%) concentrations, basal ISRs (by 34–53%), postprandial ISR AUCs (by 41–49%), and lower insulin clearance rates (by 17–22%) (all *P* < 0.05).

**Conclusions:** Fasting and postprandial plasma BCAA levels are associated with greater fasting and postprandial insulin secretion and reduced insulin clearance in healthy Chinese subjects. These observations potentially highlight an additional layer of involvement of BCAA in the regulation of glucose homeostasis.

## Introduction

The pathogenesis of type 2 diabetes mellitus (T2DM) involves not only the development of peripheral insulin resistance (i.e., decrease in insulin-mediated glucose uptake by peripheral tissues, particularly skeletal muscle), but also impaired insulin secretion from pancreatic β-cells, which leads to fasting and postprandial hyperglycemia. In fact, for as long as insulin secretion can be upregulated, insulin resistance is compensated for, and glucose homeostasis can be maintained (1). Epidemiological studies have established cross-sectional links between circulating branched-chain amino acids (BCAA) and various indices of insulin action and suggest that BCAA can predict T2DM risk (2–6). On the other hand, not many studies to date have assessed the association between circulating BCAA and robust indices of insulin secretion. In the Insulin Resistance Atherosclerosis Study (IRAS), no major links were reported between fasting BCAA levels and insulin secretion in response to an intravenous glucose challenge (7). However, a dietary protein preload augments carbohydrate-induced insulin secretion in patients with T2DM (8), whereas short-term dietary BCAA restriction reduces plasma BCAA concentrations and attenuates postprandial insulin and C-peptide concentrations after ingestion of a mixed meal in patients with obesity and T2DM (9). These observations raise the possibility that circulating BCAA, particularly in the postprandial state, affect insulin secretion or clearance, or both. We therefore examined the relationship between BCAA and insulin secretion and clearance rates in the fasting and postprandial states, assessed by mathematical modeling, in healthy Chinese subjects, i.e., a population in which pancreatic insulin secretion is particularly important in glucose homeostasis and the pathogenesis of T2DM (10). Understanding the discrete associations between BCAA and insulin turnover rates will help design interventional strategies to alleviate metabolic dysfunction in specific subsets of the population.

## Research Design and Methods

### Ethics

The study protocol was approved by the SingHealth Centralised Institutional Review Board in Singapore (CIRB Ref: 2018/2116) and was registered at clinicaltrials.gov (NCT03531879). All procedures were conducted in accordance with the Declaration of Helsinki, and all participants gave their written informed consent.

### Subjects

A total of 100 apparently healthy, middle-aged, Chinese adults with low risk of coronary heart disease participated in the study, but five subjects were excluded from the analysis because of failure to retrieve complete blood samples. Inclusion criteria were: (i) be willing and able to sign written informed consent in English or Chinese; (ii) 40–54 years old; (iii) male or female; (iv) Chinese ethnic group (having both grandparents Chinese); (v) low Framingham risk score of coronary heart disease (<10%); (vi) apparently healthy, based on clinical assessment. Exclusion criteria were: (i) food allergy to any of the constituents of the meal challenge (milk proteins, soy, lactose, including lactose intolerance); (ii) not willing or unable to comply with scheduled visits and the requirements of the protocol; (iii) pregnant or lactating women, based on clinical assessment; (iv) women on hormonal replacement therapy or contraceptives (any method and route); (v) morbid obesity (body mass index, BMI ≥ 40 kg/m^2^); (vi) previous myocardial infarction; (vii) known coronary artery disease or prior coronary revascularization; (viii) known documented peripheral arterial disease; (ix) previous stroke (defined as new focal neurological deficit persisting more than 24 h); (x) use of anti-hypertensive agents; (xi) prior history of cancer (excluding pre-cancerous lesions); (xii) life expectancy <1 year; (xiii) known definite T2DM or on treatment for T2DM, autoimmune disease or genetic disease, endocrine and metabolic diseases, including hyperlipidemia; (xiv) psychiatric illness; (xv) asthma or chronic lung disease requiring long term medications or oxygen; (xvi) chronic infective disease, including tuberculosis, hepatitis B and C; and HIV; and (xvii) currently participating or having participated in another clinical trial within 4 weeks prior to trial start.

### Experimental Design

This was a cross-sectional study conducted in two sites: screening took place at the National Heart Center Singapore (NHCS) and the metabolic testing was conducted at the Singapore Institute for Clinical Sciences (SICS), A^*^STAR. During the screening visit at NHCS, demographic and anthropometric data (date of birth, gender, weight, height, hip and waist circumference) were collected. A fasting blood sample was collected for the analysis of glucose, total cholesterol, low density lipoprotein-cholesterol (LDL-C), high density lipoprotein-cholesterol (HDL-C), and triglyceride (TG). For the metabolic testing visit, subjects were advised not to perform any strenuous activity for 2 days and to abstain from alcohol and caffeinated food and drinks for 1 day prior to being admitted to SICS. A low-fat standardized dinner was provided to subjects to be consumed by 2,200 h on the evening before the mixed meal tolerance test. Subjects arrived in the fasted state (>10 h) at SICS the following morning. After 30 min of rest, their height, weight, blood pressure, and resting heart rate were recorded, and a fasting venous blood sample was collected from an indwelling cannula inserted in the antecubital vein. Thereafter, a meal test was served and a total of nine additional blood samples were drawn at successive time points (10, 20, 30, 45, 60, 90, 120, 240, and 360 min). The composition of the test meal was based on a systematic review that defined the optimal nutritional stress test to assess phenotypic flexibility (11) and consisted of 75 g glucose, 60 g palm olein and 20 g dairy protein served in a ~337 mL liquid solution, with a total energy content of ~930 kcal (32% of energy from carbohydrate, 58% from fat, and 11% from protein).

### Sample Analyses

Plasma glucose concentration was determined by the glucose oxidase method on an automated glucose analyzer (YSI 2300 Stat Plus; YSI Life Sciences, Yellow Spring, OH). Plasma insulin and C-peptide concentrations were determined by using electrochemiluminescence technology (Roche/ Hitachi Cobas e411 immunochemistry analyzer; Roche Diagnostics, Indianapolis, IN). Plasma total TG, total cholesterol, HDL-C, LDL-C, apolipoproteins B and A1 (ApoB and ApoA1, respectively), and BCAA (leucine, isoleucine, and valine) were quantified as part of a panel of 151 different metabolomics biomarkers assessed at 0, 60, 120, 240, and 360 min by proton nuclear magnetic resonance (NMR, Nightingale Health Ltd, Helsinki, Finland) (12, 13).

## Calculations

The Homeostasis Model Assessment score of insulin resistance (HOMA-IR) was calculated as fasting insulin (mU/L) × fasting glucose (mg/dL)/405. The insulin secretion rate (ISR, in pmol/L·min) before and after ingestion of the mixed meal was assessed by using oral minimal modeling analysis of plasma C-peptide and glucose concentrations (Simulation, Analysis and Modeling Software, SAAM II version 2.3, The Epsilon Group, Charlottesville, VA), to obtain basal (fasting) and postprandial ISRs. Cumulative postprandial ISR (in pmol/L) was computed as the corresponding area under the curve (AUC) for 6 h after ingestion of the mixed meal, and insulin clearance (pools/h) was calculated by dividing insulin ISR AUC (pmol/L) by the insulin concentration AUC (pmol/L·h) (14–16).

### Statistical Analysis

All analyses were carried out with SPSS version 27 (IBM SPSS, Chicago, IL). Prior to all statistical analyses, the distributional properties of the outcome measures were evaluated for normality (Shapiro-Wilks test). Associations between BCAA and other parameters of interest were evaluated by using partial correlation analysis after adjusting for sex and BMI, and reported as partial correlation coefficients (ρ). For indices of insulin turnover (i.e., basal ISR, postprandial ISR, and clearance rate), associations were further adjusted for HOMA-IR and postprandial glucose AUC. Subjects were stratified into BCAA tertiles according to total fasting BCAA concentration and total postprandial BCAA AUC. Postprandial time courses for glucose, insulin, and C-peptide were compared across BCAA tertiles by using a linear mixed model with main effects for time and tertile, an interaction term for time-by-tertile, and covariates for sex and BMI. For outcomes without a repeated measure (i.e., glucose, insulin, and C-peptide AUCs, and HOMA-IR), comparisons across tertiles were performed by one-way analysis of variance with sex and BMI as covariates, and Šidák's *post-hoc* adjustment for multiple comparisons. Results are reported as mean ± SEM. Statistical significance was set at *P* < 0.05.

## Results

### Subject Characteristics

**Figure 1:**
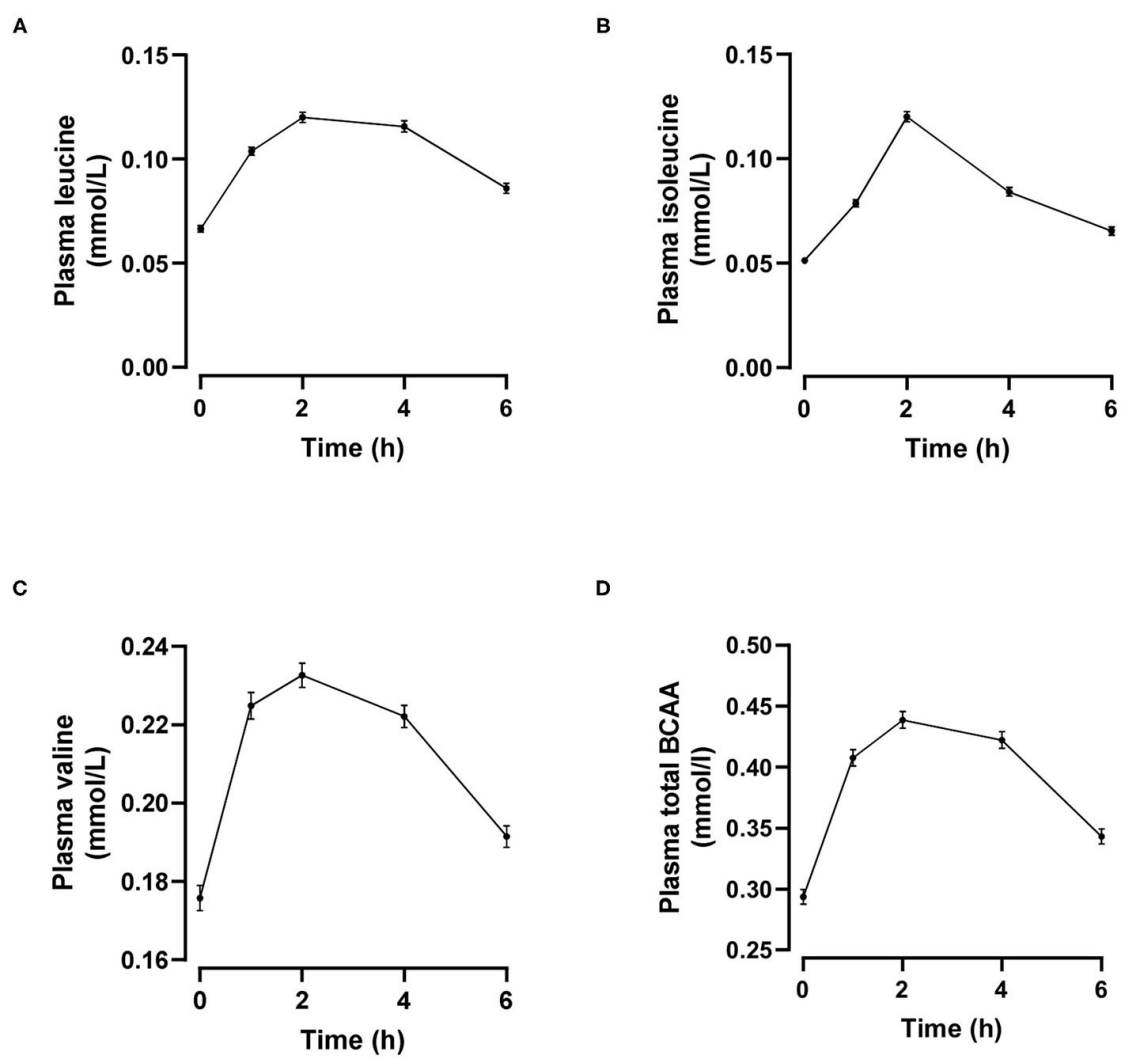
Time course of plasma leucine **(A)**, isoleucine **(B)**, valine **(C)** and total branched-chain amino acids (BCAA, **D**) after mixed meal ingestion. Data are mean ± SEM; *n* = 95.

Individual and total plasma BCAA concentrations increased from the fasting state and peaked 2 h after meal ingestion (Figure 1). Fasting BCAA concentration correlated strongly with postprandial BCAA AUC (ρ = 0.796, *P* < 0.001, *n* = 95). Subjects stratified into fasting BCAA concentration tertiles had significantly different HOMA-IR, fasting insulin, TG, HDL-C, and ApoB concentrations, and postprandial BCAA AUC, after adjusting for sex and BMI (all *P* < 0.05, Table 1). Likewise, subjects stratified into postprandial BCAA AUC tertiles had significantly different HOMA-IR, fasting insulin, TG, total cholesterol, HDL-C, LDL-C, and ApoB concentrations, and fasting BCAA levels, after adjusting for sex and BMI (all *P* < 0.05, Table 2).

**Table 1:** Anthropometric characteristics and cardiometabolic parameters of subjects stratified into fasting BCAA concentration tertiles.

	**Fasting BCAA concentration tertile**			***P***	***P*-adj**
	**1st (*n* = 30)**	**2nd (*n* = 33)**	**3rd (*n* = 32)**		
Sex (M/F)	4/26	22/11	26/6	<*0.001*	–
Age (years)	47.6 ± 0.8	47.6 ± 0.7	47.9 ± 0.7	0.955	0.762
Body mass index (kg/m^2^)	22.9 ± 0.5	23.3 ± 0.5	24.6 ± 0.5	0.058	–
Systolic BP (mmHg)	111 ± 2	116 ± 2	121 ± 2	*0.010*	0.637
Diastolic BP (mmHg)	71 ± 2	76 ± 2	79 ± 2	*0.006*	0.715
Resting heart rate (bpm)	67 ± 2	63 ± 2	67 ± 2	0.180	0.267
Fasting glucose (mg/dL)	96.0 ± 1.4	96.0 ± 1.1	98.9 ± 1.8	0.262	0.543
Fasting insulin (mU/L)	6.4 ± 0.5	6.7 ± 0.7	10.7 ± 1.1^**^^†^	<*0.001*	*0.003*
HOMA-IR	1.5 ± 0.1	1.6 ± 0.2	2.7 ± 0.3^**^^†††^	<*0.001*	*0.003*
Triglyceride (mmol/L)	0.8 ± 0.05	0.9 ± 0.07	1.5 ± 0.12^***^^†††^	<*0.001*	<*0.001*
Total cholesterol (mmol/L)	5.0 ± 0.1	5.1 ± 0.1	5.4 ± 0.1	0.138	0.107
HDL-cholesterol (mmol/L)	2.1 ± 0.1	1.9 ± 0.1	1.6 ± 0.1^*^	<*0.001*	*0.021*
LDL-cholesterol (mmol/L)	3.0 ± 0.1	3.2 ± 0.1	3.5 ± 0.1	*0.017*	0.080
ApoB (g/L)	0.71 ± 0.02	0.73 ± 0.02	0.87 ± 0.03^***^^†††^	<*0.001*	<*0.001*
ApoA1 (g/L)	1.51 ± 0.03	1.47 ± 0.03	1.43 ± 0.03	*0.033*	0.843
ApoB/ApoA1 ratio	0.46 ± 0.01	0.50 ± 0.01	0.63 ± 0.02^***^^†††^	<*0.001*	<*0.001*
Total fasting BCAA (mmol/L)	0.23 ± 0.004	0.29 ± 0.003^***^	0.36 ± 0.006^***^^†††^	<*0.001*	<*0.001*
Postprandial BCAA AUC (mmol·L^−1^·min)	126 ± 2.7	141 ± 2.1^***^	166 ± 2.2^***^^†††^	<*0.001*	<*0.001*

*Data are mean ± SEM or frequencies; n = 95. BP, blood pressure; HOMA-IR, Homeostasis Model Assessment score of insulin resistance; HDL, high-density lipoprotein; LDL, low-density lipoprotein; ApoB, apolipoprotein B; ApoA1, apolipoprotein A1; BCAA, branched chain amino acids. Statistical significance for differences between groups was determined by one-way ANOVA with sex and BMI as covariates (P-adj), followed by post-hoc pairwise comparisons with Šidák's adjustment*.

*** *P < 0.001;*

** *P < 0.01;*

* *P < 0.05 vs. 1st tertile*,

††† *P < 0.001;*

† *P < 0.05 vs. 2nd tertile. P-values < 0.05 are italicized*.

**Table 2:** Anthropometric characteristics and cardiometabolic parameters of subjects stratified into postprandial BCAA AUC tertiles.

	**Postprandial BCAA AUC tertile**			***P***	***P*-adj**
	**1st (*n* = 31)**	**2nd (*n* = 32)**	**3rd (*n* = 32)**		
Sex (M/F)	10/21	20/12	22/10	*0.007*	–
Age (years)	47.0 ± 0.8	48.5 ± 0.7	47.6 ± 0.7	0.344	0.237
Body mass index (kg/m^2^)	22.7 ± 0.5	23.4 ± 0.5	24.7 ± 0.5	*0.021*	–
Systolic BP (mmHg)	114 ± 3	116 ± 2	119 ± 2	0.205	0.781
Diastolic BP (mmHg)	73 ± 2	75 ± 1	78 ± 2	0.170	0.816
Resting heart rate (bpm)	64 ± 2	66 ± 2	66 ± 2	0.688	0.542
Fasting glucose (mg/dL)	95.6 ± 1.3	96.0 ± 1.2	99.3 ± 1.8	0.150	0.362
Fasting insulin (mU/L)	6.4 ± 0.5	6.2 ± 0.5	11.2 ± 1.2^**^^†††^	<*0.001*	<*0.001*
HOMA-IR	1.5 ± 0.1	1.5 ± 0.1	2.8 ± 0.3^**^^†††^	<*0.001*	<*0.001*
Triglyceride (mmol/L)	0.7 ± 0.1	0.9 ± 0.1	1.6 ± 0.1^***^^†††^	<*0.001*	<*0.001*
Total cholesterol (mmol/L)	5.1 ± 0.1	5.0 ± 0.1	5.5 ± 0.2^†^	*0.021*	*0.013*
HDL-cholesterol (mmol/L)	2.1 ± 0.1	1.9 ± 0.1	1.6 ± 0.1^*^^†^	<*0.001*	*0.009*
LDL-cholesterol (mmol/L)	3.1 ± 0.1	3.1 ± 0.1	3.6 ± 0.1^*^^†^	*0.003*	*0.007*
ApoB (g/L)	0.69 ± 0.02	0.73 ± 0.02	0.90 ± 0.03^***^^†††^	<*0.001*	<*0.001*
ApoA1 (g/L)	1.51 ± 0.03	1.47 ± 0.03	1.43 ± 0.03	0.152	0.980
ApoB/ApoA1 ratio	0.46 ± 0.08	0.50 ± 0.11	0.63 ± 0.11^***^^†††^	<*0.001*	<*0.001*
Total fasting BCAA (mmol/L)	0.24 ± 0.007	0.29 ± 0.007^***^	0.35 ± 0.009^***^^†††^	<*0.001*	<*0.001*
Postprandial BCAA AUC (mmol·L^−1^·min)	121 ± 1.7	143 ± 1.0^***^	167 ± 1.7^***^^†††^	<*0.001*	<*0.001*

*Data are mean ± SEM or frequencies; n = 95. BP, blood pressure; HOMA-IR, Homeostasis Model Assessment score of insulin resistance; HDL, high-density lipoprotein; LDL, low-density lipoprotein; ApoB, apolipoprotein B; ApoA1, apolipoprotein A1; BCAA, branched chain amino acids. Statistical significance for differences between groups was determined by one-way ANOVA with sex and BMI as covariates (P-adj), followed by post-hoc pairwise comparisons with Šidák's adjustment*.

*** *P < 0.001;*

** *P < 0.01;*

* *P < 0.05 vs. 1st tertile*,

††† *P < 0.001;*

† *P < 0.05 vs. 2nd tertile. P-values < 0.05 are italicized*.

### BCAA Tertiles and Meal-Induced Glucose, Insulin and C-Peptide Responses

**Figure 2:**
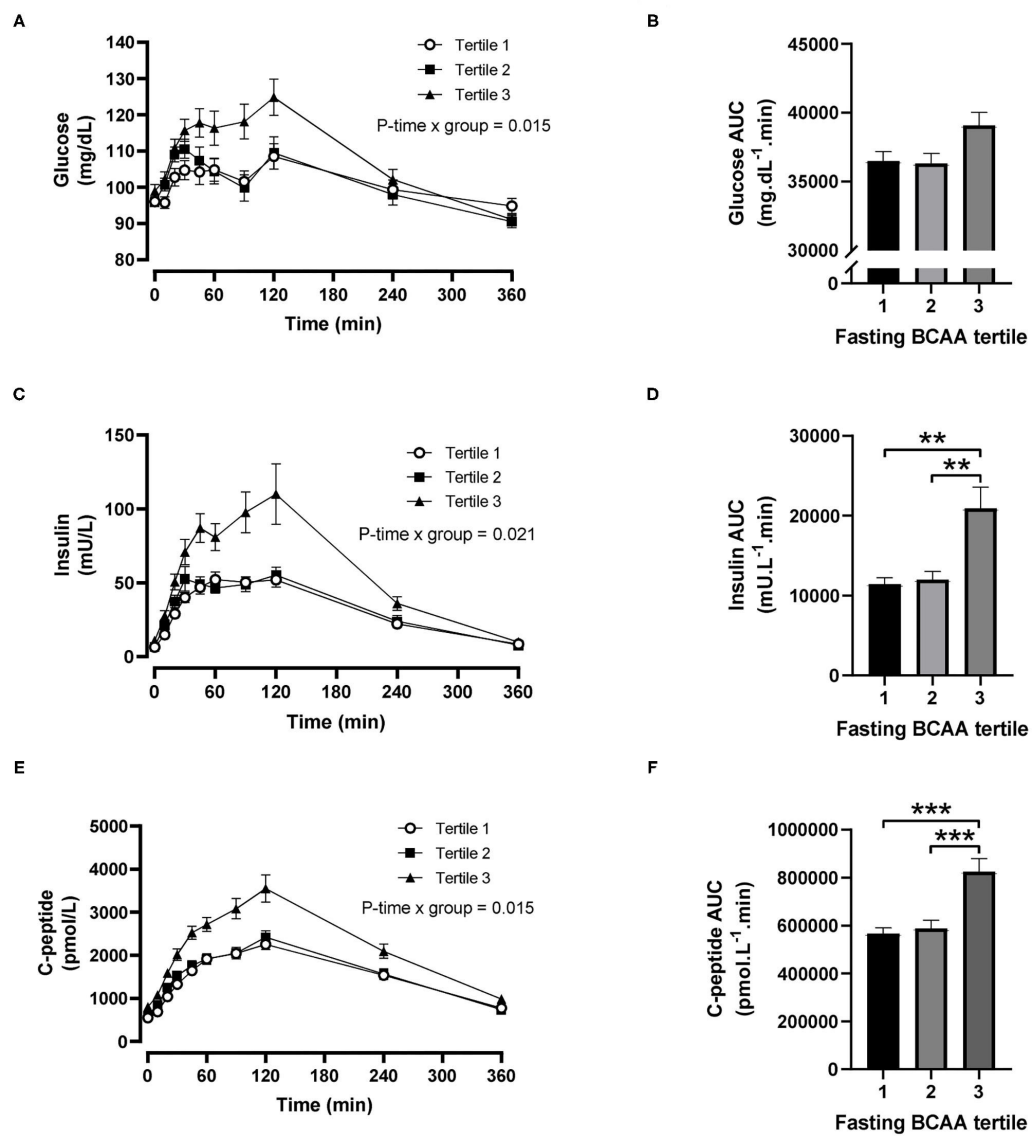
Time courses and areas under the curve (AUC) of plasma glucose, insulin and C-peptide after ingestion of a mixed meal in subjects stratified in fasting branched-chain amino acid (BCAA) concentration tertiles. Glucose **(A,B)**, insulin **(C,D)** and C-peptide **(E,F)** concentrations (linear mixed model) and AUCs (one-way ANOVA) after ingestion of a liquid mixed meal by fasting BCAA concentration tertiles. Data are mean ± SEM; *n* = 30–33 per group. All analyses have been adjusted for sex and BMI. Corresponding values are significantly different between groups (Šidák-corrected): **P < 0.01, ***P < 0.001.

For fasting BCAA tertiles, there were significant time-by-tertile interactions for plasma glucose (*P* = 0.015), insulin (*P* = 0.021) and C-peptide (*P* = 0.015) concentrations (Figures 2A,C,E). The AUCs for plasma glucose were significantly different overall (*P* = 0.030) but pairwise comparisons did not reach statistical significance after Šidák's adjustment (*P* = 0.057 for tertile 1 vs. 3 and *P* = 0.069 for tertile 2 vs. 3; Figure 2B). The AUCs for insulin and C-peptide were both significantly greater in the third BCAA tertile compared with the first and second BCAA tertiles (all *P* < 0.004; Figures 2D,F).

**Figure 3:**
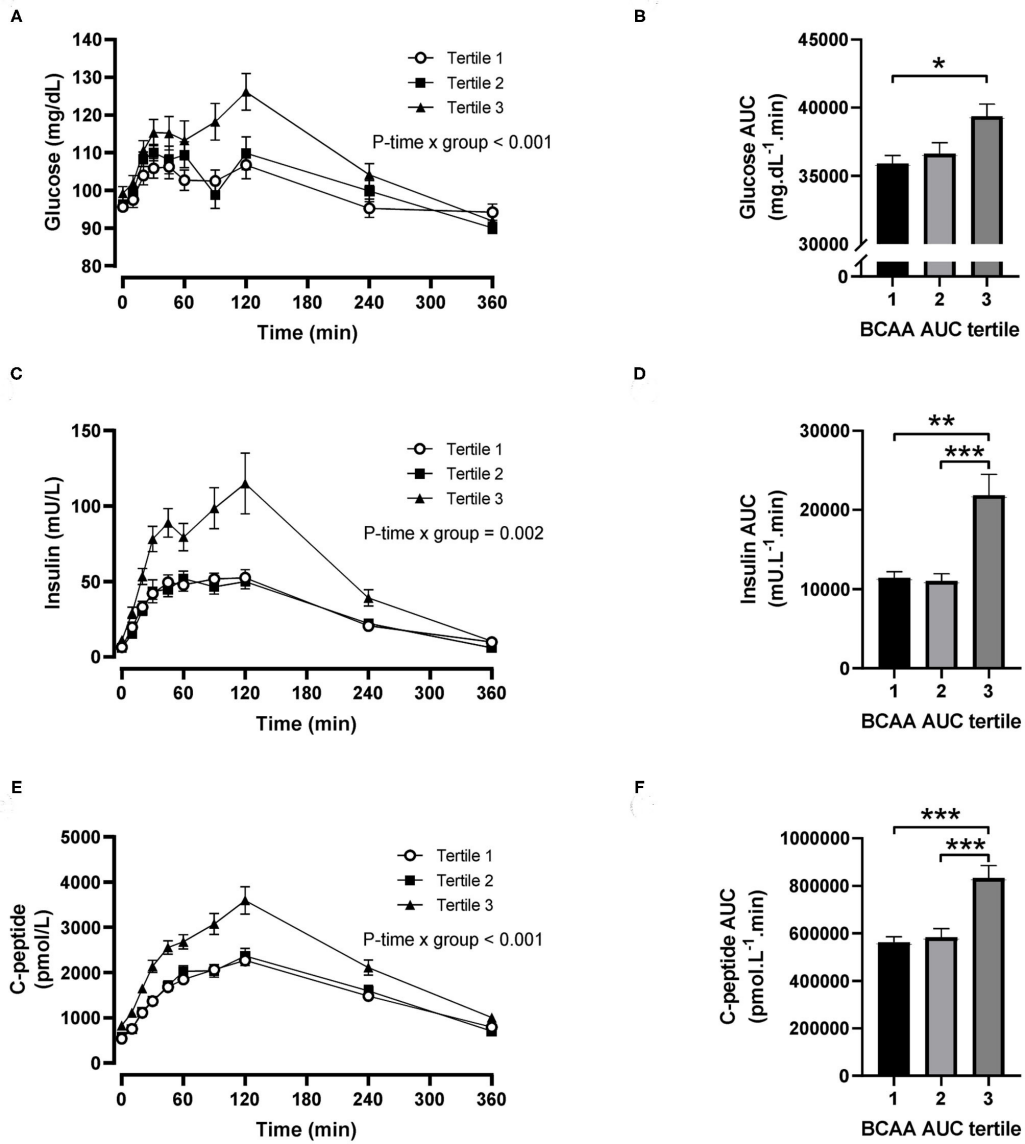
Time courses and areas under the curve (AUC) of plasma glucose, insulin and C-peptide after ingestion of a mixed meal in subjects stratified in postprandial branched-chain amino acid (BCAA) AUC tertiles. Glucose **(A,B)**, insulin **(C,D)** and C-peptide **(E,F)** concentrations (linear mixed model) and AUCs (one-way ANOVA) after ingestion of a liquid mixed meal by postprandial BCAA AUC tertiles. Data are mean ± SEM; *n* = 31–32 per group. All analyses have been adjusted for sex and BMI. Corresponding values are significantly different between groups (Šidák-corrected): *P < 0.01, **P < 0.05, ***P < 0.001.

For postprandial BCAA tertiles, there were significant time-by-tertile interactions for plasma glucose (*P* < 0.001), insulin (*P* = 0.002) and C-peptide (*P* < 0.001) concentrations (Figures 3A,C,E). The AUC for plasma glucose was significantly greater in the third BCAA tertile compared with the first BCAA tertile (*P* = 0.021; Figure 3B), and the AUCs for insulin and C-peptide were both significantly greater in the third BCAA tertile compared with the first and second BCAA tertiles (all *P* < 0.002; Figures 3D,F).

### Association Between BCAA and Insulin Metabolism

**Figure 4:**
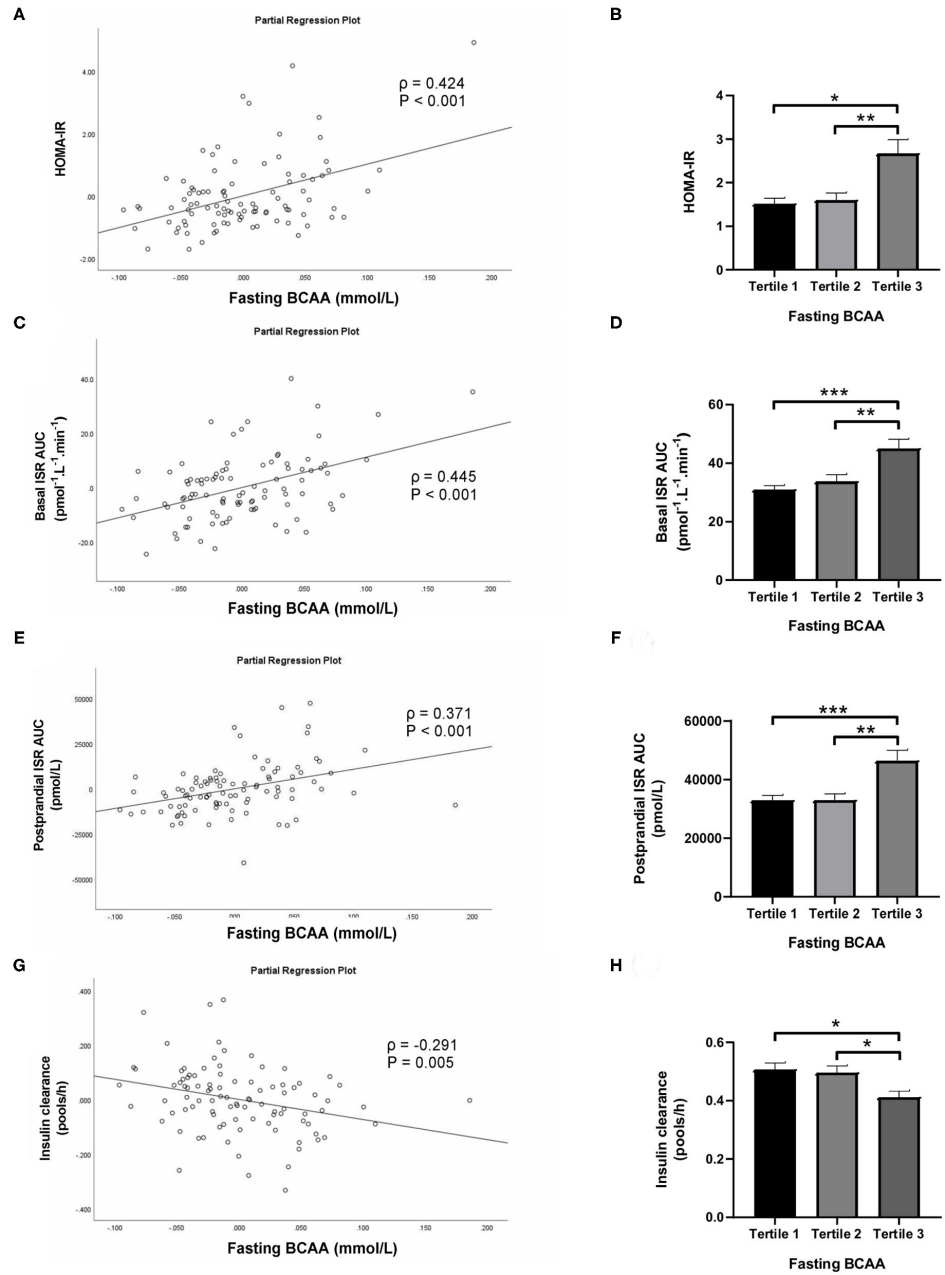
Homeostasis Model Assessment of Insulin Resistance (HOMA-IR; **A,B**), basal insulin secretion rate (ISR; **C,D**), total postprandial ISR area under the curve (AUC; **E,F**) and insulin clearance **(G,H)** in relation to fasting BCAA concentration (left) and in subjects stratified to tertiles of fasting BCAA concentration (right). Data are mean ± SEM; *n* = 30–33 per group. Continuous analyses are depicted as partial correlation plots and differences among tertiles are determined by one-way ANOVA. All analyses have been adjusted for sex and BMI. Corresponding values are significantly different between groups (Šidák-corrected): *P < 0.05, **P < 0.01, ***P < 0.001.

**Figure 5:**
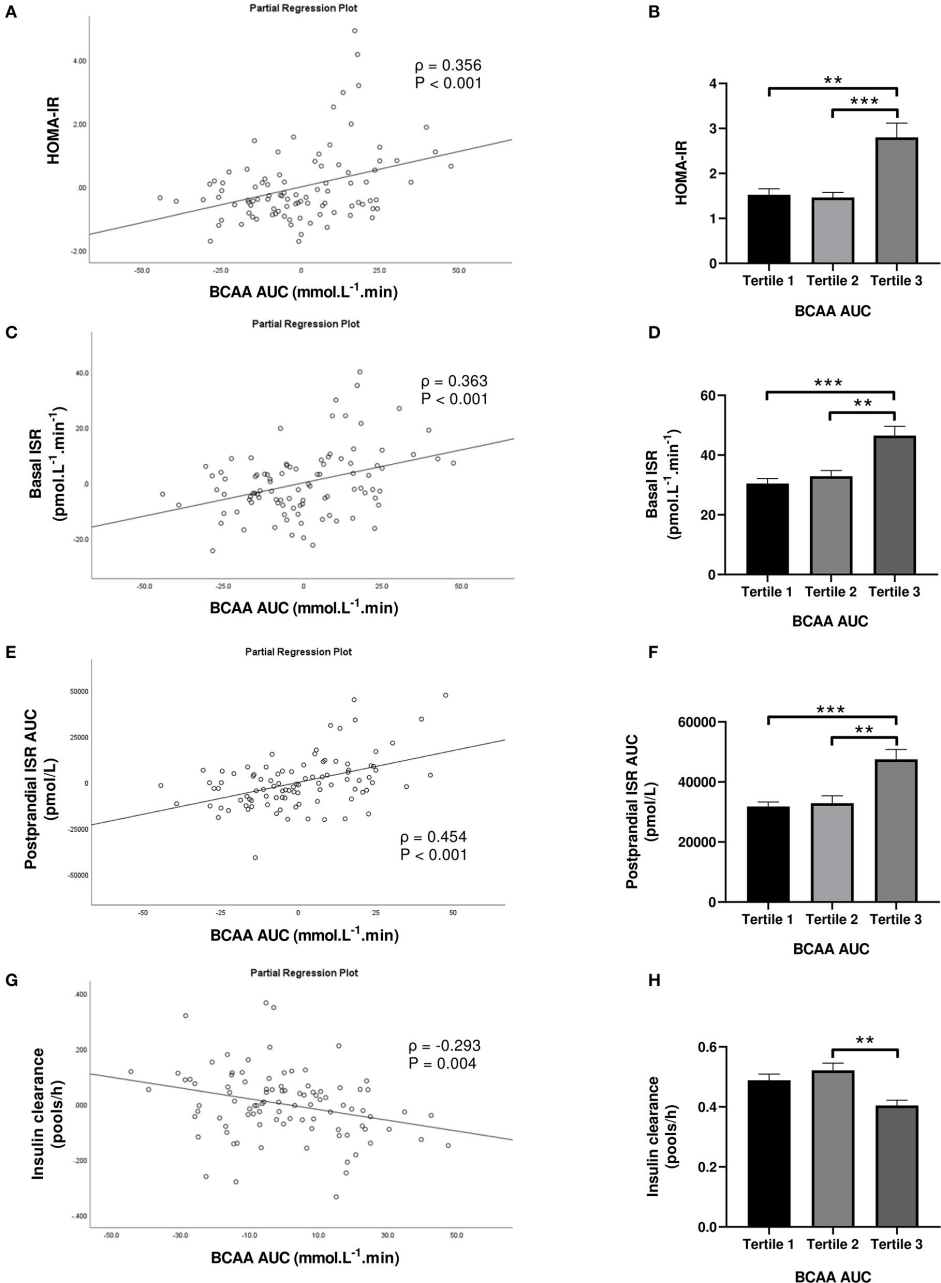
Homeostasis Model Assessment of Insulin Resistance (HOMA-IR; **A,B**), basal insulin secretion rate (ISR; **C,D**), total postprandial ISR area under the curve (AUC; **E,F**) and insulin clearance **(G,H)** in relation to postprandial BCAA AUC (left) and in subjects stratified to tertiles of postprandial BCAA AUC (right). Data are mean ± SEM; *n* = 31–32 per group. Continuous analyses are depicted as partial correlation plots and differences among tertiles are determined by one-way ANOVA. All analyses have been adjusted for sex and BMI. Corresponding values are significantly different between groups (Šidák-corrected): **P < 0.01, ***P < 0.001.

Subjects in the third tertile of fasting BCAA concentration (Figure 4) and BCAA AUC (Figure 5) had significantly greater HOMA-IR score, basal ISR, and postprandial ISR AUC, and significantly lower insulin clearance compared with subjects in the first and second tertiles (all *P* < 0.05, after adjustment for BMI and sex—only the difference in insulin clearance between postprandial BCAA AUC tertile 3 and 1 did not reach significance, *P* = 0.108).

When fasting BCAA concentration and postprandial BCAA AUC were treated as continuous variables (*n* = 95), fasting BCAA concentration (Figure 4) was positively associated with HOMA-IR (ρ = 0.424, *P* < 0.001), basal ISR (ρ = 0.445, *P* < 0.001) and postprandial ISR AUC (ρ = 0.371, *P* < 0.001), and negatively with insulin clearance (ρ = −0.291, *P* = 0.005). Similarly, postprandial BCAA AUC (Figure 5) was positively associated with HOMA-IR (ρ = 0.356, *P* < 0.001), basal ISR (ρ = 0.363, *P* < 0.001) and postprandial ISR AUC (ρ = 0.454, *P* < 0.001), and negatively with insulin clearance (ρ = −0.293, *P* = 0.004). Upon further adjustment of the models for HOMA-IR and postprandial glucose AUC, the associations of fasting and postprandial BCAA with basal ISR, postprandial ISR AUC, and insulin clearance continued to hold their statistical significance (all *P* < 0.05), except for the association between postprandial BCAA AUC and basal ISR which only approached significance (*P* = 0.075).

## Discussion

In the early stages of the pathogenesis of T2DM, peripheral insulin sensitivity is decreased, but whether or not this will result in an overt disruption of glucose homeostasis largely depends on pancreatic insulin response: functioning β-cells have the ability to upregulate insulin secretion and compensate for insulin resistance and thereby maintain glucose homeostasis. Chronic hypersecretion of insulin, however, can lead to a decline in β-cell mass and function and subsequently, to reduced insulin secretion which allows hyperglycemia to manifest (17). It is also possible that hyperinsulinemia in itself contributes to insulin resistance, which then sets up a vicious cycle that predisposes to T2DM (18–21). In this study of healthy Chinese men and women, we documented significant positive associations between circulating BCAA in the fasting and postprandial states and the concentrations of glucose and insulin in response to mixed meal ingestion, and the rates of fasting and postprandial pancreatic insulin secretion, and significant but weaker negative associations with insulin clearance. The tertile analysis further suggested these relationships are characterized by a threshold, J-like association, rather than a linear dose-response.

The BCAAs leucine, isoleucine and valine play a critical role in β-cell signaling and metabolism, acutely stimulating insulin secretion and activating the regulatory serine/threonine kinase mammalian target of rapamycin complex 1 (mTORC1), a kinase that promotes increased β-cell mass and function (22, 23). The role of BCAAs in metabolic health is controversial—increasing dietary BCAA intake has been linked with various beneficial metabolic effects (e.g., increased satiety, protein synthesis, and diet-induced thermogenesis), but paradoxically circulating BCAA levels have been associated with obesity, dyslipidemia, insulin resistance and T2DM (2, 4–6, 24). It is postulated that chronic hyperactivation of mTORC1 by BCAA can inhibit insulin/growth factor signaling, through ribosomal protein S6 kinase (S6K)-mediated inhibitory phosphorylation of the insulin receptor substrate (4). In turn, decreasing insulin sensitivity increases demand on β-cells to produce more insulin for adequate disposal of circulating glucose, and maintenance of glucose homeostasis. Insulin resistance, or BCAA themselves, may also lead to a reduction in insulin clearance, which can further augment availability of circulating insulin (7). Reductions in insulin clearance rates may be the result of reduced hepatic and extrahepatic extraction (25). Chronic hyperinsulinemia, in concurrence with other factors such as lipotoxicity and increased islet amyloid polypeptide secretion (26, 27), eventually leads to islet dysfunction-characterized by an initial increase in β-cell proliferation and islet mass, followed by apoptosis-leading to the onset of T2DM (4). Furthermore, chronic endoplasmic reticulum stress in β-cells in the face of obesity and insulin resistance may lead to increased Large neutral Amino Acid Transporter 1 (LAT1) expression, a transporter of BCAA into β-cells. This can further augment mTORC1 activation and result in amplification of the compensatory increase in β-cell function and mass, hastening the path to β-cell apoptosis and failure (28).

Here we show that fasting and postprandial plasma BCAA levels in healthy subjects of Chinese descent are associated with greater basal and postprandial insulin secretion by pancreatic β-cells (assessed by mathematical modeling of glucose and C-peptide concentrations using the oral minimal model) and reduced insulin sensitivity (assessed by the HOMA-IR score). Although our study cannot establish cause-and-effect relationships, our findings may help explain the observation by Chen et al. (3) in which higher fasting plasma BCAA levels predicted 10-year risk of developing T2DM in a Chinese population. The reported associations between BCAA and various T2DM-related outcomes may vary by ethnicity. In the IRAS, plasma BCAAs were associated with incident T2DM and underlying metabolic abnormalities (such as the acute insulin response to intravenous glucose and the metabolic clearance rate of insulin) to a greater extent in Caucasians, and maybe also in Hispanics, than in African Americans (7). Also, in a 19-year follow-up study of a non-diabetic British population, stronger adverse associations were observed between BCAA and incident diabetes in South Asian than in Caucasian men (29). However, Tai et al. (30) observed similar differences in a variety of amino acids (including the BCAAs) between high and low tertiles of insulin resistance in a group of Chinese and Asian-Indian individuals, suggesting there are at least some physiological similarities in the relationship between BCAA availability and insulin action across ethnicities. Differences in plasma BCAA levels may therefore explain part of the ethnic differences in the development of insulin resistance and T2DM. It has been hypothesized that an increase in plasma BCAA in states of insulin resistance occurs as a result of increased protein degradation, independent of dietary intake (4). Our observation of a tight positive correlation between fasting plasma BCAA concentration and postprandial BCAA AUC is consistent with this assertion and suggests that protein degradation and reduced catabolism of BCAA, rather than increased dietary intake, are central to raising plasma BCAA, which may then lead to increased insulin secretion.

A key finding from our tertile analyses is that fasting and postprandial plasma BCAAs appear to be similar in their ability to resolve different levels of glucose tolerance, insulin resistance, and insulin secretion and clearance. This is consistent with the observation that BCAA excursions during an oral glucose tolerance test are not an independent predictor of glucose tolerance, insulin sensitivity and β-cell function among mostly non-Asian adolescents with obesity (31). The somewhat better resolution with postprandial than fasting BCAA tertiles for some metabolic outcomes (e.g., better discrimination between tertiles for postprandial glucose AUC, and fasting LDL-C, HDL-C and ApoB concentrations), but certainly not all (i.e., insulin clearance), could be due to the dynamic nature of metabolism after meal ingestion compared with the static nature of fasting assessments. However, these differences are unlikely to be clinically significant and thus do not justify the additional burden of measuring postprandial BCAA for predictive purposes. The fasting plasma BCAA concentration is an equally good biomarker, particularly since it was tightly correlated with postprandial BCAA AUC, and the relationship of circulating BCAA with metabolic function appears to be threshold-dependent. These findings mirror the observation by Tricò et al. (31) of greater BCAA (valine, leucine and isoleucine) concentrations in the fasting state and after an oral glucose challenge in the most insulin-resistant tertile of their subjects compared with the other two tertiles (middle and most insulin-sensitive, who had similar values). Likewise, our subjects in the highest tertile for fasting or postprandial BCAA had 7–10% greater postprandial glucose concentrations, 74–98% greater postprandial insulin concentrations, 67–91% greater HOMA-IR scores, 34–53% greater basal ISRs, 41–49% greater postprandial ISR AUCs, and 17–22% lower insulin clearance rates, compared with subjects in the other two tertiles who had very similar values. The associations between insulin secretion and clearance rates and fasting and postprandial BCAA largely persisted even after further adjustments for HOMA-IR and postprandial glucose AUC. In agreement with our findings, Karusheva et al. (9) found that severe reduction (60%) of dietary BCAA intake over alternate weeks for 1 month led to a 17% decrease in fasting plasma BCAA concentrations and a 28% reduction in postprandial insulin concentrations in patients with obesity and T2DM (9). Similarly, consumption of isocaloric protein-restricted diets (7–9% of total energy from protein) for 1–2 months led to a ~15% decrease in fasting plasma BCAA and was associated with reductions in fasting glucose in overweight men (32). Given that subjects in our study were non-diabetic and had—on average—half the fasting plasma concentrations of BCAA of the T2DM subjects in Karusheva et al. (9) and the overweight subjects in Fontana et al. (32), it remains to be seen if a relatively modest reduction in dietary BCAA intake (which would be easier to adhere to for longer periods of time) could result in significant improvements in metabolic function in at-risk individuals.

Our study cannot establish cause-and-effect relationships, so the observed associations may not necessarily be due to BCAA directly affecting insulin metabolism. For instance, studies have shown that exercise training (which increases insulin sensitivity) has little effect on plasma BCAA concentrations (33, 34). Still, the decrease in the molar sum of circulating BCAA was the best metabolic predictor of training-induced improvements in insulin sensitivity in overweight, insulin-resistant subjects (34). Similar observations for indices of insulin secretion and clearance are lacking. Higher levels of circulating glycine in lean individuals and the increase in plasma glycine in overweight individuals after exercise training (along with altered levels of several urinary glycine adducts and other metabolites), suggest that exercise facilitates elimination of excess acyl groups derived from BCAA and aromatic amino acid metabolism (34). Therefore, the fact that exercise training increases insulin sensitivity but does not consistently decrease circulating BCAA does not necessarily preclude that BCAA are involved in the pathogenesis of insulin resistance, or modulate insulin secretion and clearance.

Elevated plasma BCAA were also associated with an unfavorable fasting blood lipid profile in our subjects, including higher TG, LDL-C and ApoB/ApoA1 ratio and lower HDL-C, consistent with the BCAA-dyslipidemia links established in Asian and Caucasian populations (35–38). In two of these studies, BCAAs remained significantly associated with a more adverse lipid profile even when adjusted for impaired glucose metabolism, suggesting that BCAA may be an independent cardiovascular disease risk factor (35, 37). In support, it had been previously shown in murine models that a diet deficient in BCAA independently reduces circulating TG and total cholesterol levels (39). Taken together, these data suggest a potential role of BCAA in regulating lipid metabolism, which should be further explored regarding its contribution to the development of insulin resistance and its metabolic sequelae (40, 41).

Our study has a number of limitations. First, we did not obtain food records to assess how habitual dietary BCAA intake might have affected BCAA concentrations. We were also not able to discern between the contribution of exogenous (from dietary intake) and endogenous (from protein degradation in peripheral tissues) sources to fasting and postprandial plasma BCAA levels without the use of amino acid tracers. A better understanding of the contribution of dietary vs. catabolic pathways to circulating BCAA will help design effective interventions to modulate BCAA availability and thereby insulin secretion and clearance, which can potentially improve metabolic function. Intervention-led reductions of insulinemia have been associated with improvements in various metabolic outcomes (42–44), and BCAAs have been identified as a modifiable nutrient target with the potential to ameliorate metabolic dysfunction (2, 4, 9, 32, 45). Nevertheless, since our observations were made in healthy, non-diabetic subjects of Chinese descent, for the time being they cannot be generalized to other ethnicities or patients with T2DM. Last, but not least, since tertiles are specific to this group of subjects, we cannot interpret or generalize our results in terms of absolute cut-offs for fasting and postprandial BCAA levels.

In conclusion, our observations suggest a possible involvement of circulating BCAA in the regulation of insulin secretion and clearance, and thereby glucose homeostasis. Accordingly, the role for dietary plasma BCAA reduction as a nutritional strategy to improve metabolic health should be explored in future studies, and take into account obesity, insulin sensitivity, ethnicity, and prevailing levels of circulating BCAAs. Further studies will also be needed to confirm the clinical utility of plasma BCAA reduction threshold for at-risk individuals.

## Data Availability Statement

The raw data supporting the conclusions of this article will be made available by the authors, by request, without undue reservation.

## Ethics Statement

This study involving human participants were reviewed and approved by SingHealth Centralised Institutional Review Board, Singapore (CIRB Ref: 2018/2116). The participants provided their written informed consent to participate in this study.

## Author Contributions

CD: conceptualization, methodology, validation, formal analysis, investigation, resources, writing - original draft, visualization, and project administration. LE, NB, LS, HG, and JY: investigation, writing – review & editing, and project administration. KY, LA-G, and ML: writing – review & editing, supervision, project administration, and funding acquisition. FM: conceptualization, methodology, validation, formal analysis, writing – review & editing, supervision, project administration, and funding acquisition. All authors contributed to the article and approved the submitted version.

## Conflict of Interest

CD, NB, LE, and LA-G are (or have been, at the time of the study) employees of Nestlé Research, Singapore. The remaining authors declare that the research was conducted in the absence of any commercial or financial relationships that could be construed as a potential conflict of interest.

## Abbreviations

ApoA1: apolipoprotein A1
ApoB: apolipoprotein B
AUC: area-under-the-curve
BCAA: branched chain amino acid
BMI: body mass index
HDL-C: high-density lipoprotein cholesterol
HOMA-IR: homeostatic model assessment of insulin resistance
LDL-C: low-density lipoprotein cholesterol
T2DM: type 2 diabetes mellitus
TG: triglyceride.
